# Dynorphin 1-17 and Its N-Terminal Biotransformation Fragments Modulate Lipopolysaccharide-Stimulated Nuclear Factor-kappa B Nuclear Translocation, Interleukin-1beta and Tumor Necrosis Factor-alpha in Differentiated THP-1 Cells

**DOI:** 10.1371/journal.pone.0153005

**Published:** 2016-04-07

**Authors:** Siti Sarah Fazalul Rahiman, Michael Morgan, Paul Gray, Paul Nicholas Shaw, Peter John Cabot

**Affiliations:** 1 School of Pharmacy, The University of Queensland, Brisbane, QLD, Australia; 2 School of Pharmaceutical Sciences, Universiti Sains Malaysia, Minden, Penang, Malaysia; 3 Institute for Molecular Bioscience, The University of Queensland, Brisbane, QLD, Australia; 4 School of Medicine, The University of Queensland, Brisbane, QLD, Australia; 5 Department of Anaesthesia, Princess Alexandra Hospital, Brisbane, QLD, Australia; Temple University School of Medicine, UNITED STATES

## Abstract

Dynorphin 1–17, (DYN 1–17) opioid peptide produces antinociception following binding to the kappa-opioid peptide (KOP) receptor. Upon synthesis and release in inflamed tissues by immune cells, DYN 1–17 undergoes rapid biotransformation and yields a unique set of opioid and non-opioid fragments. Some of these major fragments possess a role in immunomodulation, suggesting that opioid-targeted therapeutics may be effective in diminishing the severity of inflammatory disorders. This study aimed to examine the immunomodulatory effects of DYN 1–17 and major N-terminal fragments found in the inflammatory environment on nuclear factor-kappaB/p65 (NF-κB/p65) nuclear translocation and the release of interleukin-1beta (IL-1β) and tumor necrosis factor-alpha (TNF-α) from lipopolysaccharide (LPS)-stimulated, differentiated THP-1 cells. The results demonstrate that NF-κB/p65 nuclear translocation was significantly attenuated following treatment with DYN 1–17 and a specific range of fragments, with the greatest reduction observed with DYN 1–7 at a low concentration (10 nM). Antagonism with a selective KOP receptor antagonist, ML-190, significantly reversed the inhibitory effects of DYN 1–17, DYN 1–6, DYN 1–7 and DYN 1–9, but not other DYN 1–17 N-terminal fragments (DYN 1–10 and 1–11) on NF-κB/p65 nuclear translocation. DYN 1–17 and selected fragments demonstrated differential modulation on the release of IL-1β and TNF-α with significant inhibition observed with DYN 1–7 at low concentrations (1 nM and 10 pM). These effects were blocked by ML-190, suggesting a KOP receptor-mediated pathway. The results demonstrate that DYN 1–17 and certain N-terminal fragments, produced in an inflamed environment, play an anti-inflammatory role by inhibiting NF-κB/p65 translocation and the subsequent cytokine release through KOP receptor-dependent and independent pathways.

## Introduction

Dynorphin 1–17 (DYN 1–17) opioid peptide is endogenously released from immunocytes and neurons upon enzymatic cleavage of the precursor prodynorphin molecule, yielding a 17-amino acid sequence. It selectively binds to the kappa-opioid receptor (KOP receptor) and may also interact with delta-opioid receptor (DOP receptor) and to a lesser extent at mu-opioid receptor (MOP receptor) [1–3]. Similarly to other endogenous opioids, DYN 1–17 is susceptible to rapid enzymatic degradation, generating a variety of smaller fragments representing a number of cleavage points [4]. Our laboratory has previously demonstrated that DYN 1–17 is biotransformed in rat inflamed tissue to a unique array of opioid and non-opioid fragments, different to those seen within non-inflamed tissue or blood at acidic pH, reflecting the naturally occurring inflammatory environment [5]. In addition to its analgesic effect, evidence has also reported the involvement of DYN 1–17 and its major biotransformation fragments in the regulatory control of both cellular and humoral immune responses [6, 7] including enhancement of the tumouricidal activity of murine macrophages upon stimulation by lipopolysaccharide (LPS) and interferon-gamma (IFN-γ) [8], zymosan-mediated oxidative burst in the macrophage cell line J774 [9] and suppression of the natural killer cell and antigen-specific cytolytic T cell activity [10]. The underlying signal transduction mechanisms required for these activities, however, remain poorly understood.

The nuclear factor-kappa B (NF-κB) signalling pathway is one of the possible mechanisms that mediates the immunomodulatory roles of opioids in inflammation and is central to the pathogenesis of a range of chronic inflammatory-related diseases [11]. Under resting cell conditions, inactive, cytoplasmic NF-κB is bound to its inhibitor proteins, the IκBs. Activation of IκB kinase (IKK) complexes by pro-inflammatory cytokines e.g. tumor necrosis factor-alpha (TNF-α) or LPS leads to phosphorylation of IκB and proteasome degradation, releasing active NF-κB complexes in the cytoplasm. Activated NF-κB complexes translocate to the nucleus, then bind to the DNA promoter region followed by transcription and expression of various inflammatory mediators such as cytokines, chemokines, and endothelial cell adhesion molecules [12, 13]. Activation of the NF-κB signalling pathway provides a positive autoregulatory loop in synchronizing and maintaining immune response towards infection or painful stimuli [14]. For instance, NF-κB activation of the innate immune system triggers the release of pro-inflammatory cytokines and subsequently these mediators further promote the infiltration and migration of immunocytes in the adaptive immune system to coordinate the inflammatory response to produce pain signals [15]. The MOP receptor agonist, morphine, has been reported to have differential modulation on LPS-induced activation of NF-κB and downstream release of TNF-α and IL-6 in macrophages, depending on the morphine concentrations [16] whereas in another study, this opioid peptide was found to inhibit NF-κB translocation in T cells. The discrepancy might be due to that the MOP receptor is coupled to different second messenger systems in different cell types, causing cell type-specific effects of the morphine [17]. These findings, however, suggest an association between opioids and NF-κB pathway in modulating the severity of inflammatory disorders. In this study, we examine the immunomodulatory effects of DYN 1–17 and major N-terminal fragments observed in the inflammatory environment, on LPS-stimulated NF-κB/p65 nuclear translocation and downstream release of inflammatory cytokines (IL-1β and TNF-α) in THP-1 cells and elucidate the involvement of the KOP receptor in this pathway. Our results reveal a potential role for DYN 1–17 biotransformed fragments in mediating inflammatory signals in macrophages through KOP receptor pathways.

## Materials and Methods

### Reagents

DYN 1–17, and a range of N-terminal fragments (DYN 1–6, DYN 1–7, DYN 1–9, DYN 1–10 and DYN 1–11) were supplied by Mimotopes Pty. Ltd, Australia. Purity was greater than 95% for all peptides. LPS from *Escherichia coli*, serotype O55:B5 was purchased from Enzo Life Sciences (Farmingdale, NY, USA), phorbol 12-myristate 13-acetate (PMA) was purchased from Adipogen (San Diego, CA, USA) and 4',6-diamidino-2-phenylindole (DAPI) was purchased from Cayman Chemical (MI, USA). Primary rabbit monoclonal anti-NF-κB/p65 [E379] antibody (ab32536), goat anti-rabbit IgG H&L (Alexa Fluor 555; ab150078) and goat anti-mouse IgG H&L (Alexa Fluor 647; ab150115) were provided by Abcam (Cambridge, MA, USA). The 3-(4,5-dimethyl-2-thiazolyl)-2,5-diphenyl-2H-tetrazolium bromide (MTT) reagent, dimethyl sulfoxide (DMSO), IMD-0354 and foetal bovine serum (FBS) were purchased from Sigma Aldrich (St Louis, MO, USA) whereas penicillin-streptomycin (Pen-Strep) solution and RPMI 1640 medium were supplied by Life Technologies (VIC, Australia). Primary mouse monoclonal KOP receptor antibody was obtained from Life Research Pty. Ltd., Australia.

### Cell culture and differentiation

The human monocytic cell line, THP-1 (ATCC No TIB-202) cells were grown in suspension for up to 30 passages and maintained in culture medium containing RPMI 1640 supplemented with 10% FBS, 2 mM L-glutamine, 2 g/L D-glucose and 1% Pen-Strep in a humidified atmosphere of 5% CO_2_ at 37°C [18, 19]. The induction of THP-1 cells into mature macrophage-like state was achieved in all experiments by re-suspending the cells in culture medium containing PMA (50 nM), for 48 hr. Stimulation with PMA has been shown to induce the differentiation of THP-1 cells into functional macrophages [20].

### Assessment of THP-1 cells viability

The viability of THP-1 cells was determined by performing an MTT assay as described previously [21, 22]. Briefly, PMA-induced THP-1 cells were seeded in 24 well plates (5 x 10^5^ cells/well) and cultured in 1 mL of culture medium containing DYN 1–17 and selected fragments at different concentration (10 nM and 1 μM) for 24 hr at 37°C under 5% CO_2_. Cells incubated with culture medium alone served as the positive control for this assay. MTT reagent (220 μL/well) was then added to the cell cultures to reach a final concentration of 2.5 mg/mL and incubated for an additional 2 hr at 37°C. The dark crystals formed were dissolved by adding DMSO (1200 μL) into each well at 37°C. Subsequently, the plate was incubated with gentle continuous agitation for 20 minutes on an orbital shaker (Ratek Instruments, VIC, Australia). Aliquots (100 μL) from each well were then transferred in duplicate into a 96-well plate. Wells containing DMSO only in equivalent volume were used as blank. The optical density of the produced intracellular formazan, proportional to the number of viable cells present, was measured by using a microplate reader (Bio-Rad Laboratories, CA, USA) at 590 nm. The average viable cell value after blank absorbance subtraction was calculated as a fraction of the average value obtained from cells incubated with medium alone.

### Assessment of LPS-stimulated NF-κB/p65 translocation in differentiated THP-1 cells

Cells were seeded in 96-well black microplates at a density of 1.5 x 10^5^cells (150 μL/well) and induced by PMA (50 nM) for 48 hr followed by a 24-hour incubation in PMA-free culture medium. After differentiation into macrophages, cells were serum-starved in 2% FBS-containing RPMI 1640 medium for 15 hr before the medium was discarded. LPS (1 μg/mL) was used to stimulate the differentiated THP-1 cells whereas non-stimulated cells received an equivalent volume of the media, serving as the negative control. To check the functionality of our NF-κB/p65 translocation assay, a synthetic selective NF-κB inhibitor, IMD-0354 (10 μM; Sigma-Aldrich, MO, USA) was used to treat both LPS- and non-stimulated cells for an hour in a constant environment of 5% CO_2_ at 37°C. The corresponding DMSO concentration (0.1% v/v) was similarly prepared as a diluent control for IMD-0354.

### Treatment of DYN 1–17 and selected fragments

Differentiated THP-1 cells (1.5 x 10^5^cells) were stimulated with LPS as previously described in the presence of DYN 1–17 and other fragments at two different concentrations (10 nM and 1 μM) for an hour in a humidified atmosphere at 37°C and 5% CO_2_. Different concentrations of the DYN 1–17 and the fragments were prepared by diluting the stock solutions in RPMI 1640 supplemented with 10% FBS. LPS-stimulated and non-stimulated groups were prepared as described previously. The KOP receptor activities of DYN 1–17 and other fragments on NF-κB/p65 translocation were determined using ML-190, a selective KOP receptor antagonist. The cells were pre-treated with ML-190 (1 μM) for an hour followed by co-treatment with DYN 1–17 fragments (10 nM) and ML-190 (1 μM) for an additional hour at 37°C under 5% CO_2_. Co-treatment of IMD-0354 (10 μM) and ML-190 (1 μM) was also performed separately to confirm that there was no cross-activity between both drugs. A corresponding DMSO concentration (0.1% v/v) was similarly prepared as a diluent control for both IMD-0354 and ML-190.

### Assessment of NF-κB/p65 translocation and image analysis

Differentiated THP-1 cells receiving treatment, both non-stimulated and LPS-stimulated were fixed with freshly prepared cold formaldehyde (3.7%) for 15 minutes and then rinsed three times with PBS before adding blocking buffer (containing 5% normal goat serum and 0.3% Triton X-100) for an hour at room temperature to allow cell permeabilisation and to block non-specific protein-protein interactions. Primary anti-NF-κB/p65 rabbit monoclonal antibody (1:400) was then added into each well and incubated overnight at 4°C. Next, the cells were incubated with secondary Alexa Fluor^®^ 555 goat anti-rabbit IgG (H+L) polyclonal antibody (1:500) for 2 hr followed by DAPI (2 μg/mL) staining for nucleic acid detection for 10 minutes at room temperature under subdued laboratory lighting. Cells were washed with PBS in triplicates between the incubations. The experimental plates were imaged using the ImageXpress Micro high content screening imaging system (Molecular Devices, Sunnyvale, CA). Four fields of view (2000 pixel × 2000 pixel each) were captured from each well with a 10x-magnifiying lens. Images were analyzed using customised colocalisation algorithms provided in the Metamorph software. NF-κB/p65 immunofluorescence derived from the images at the nuclear level, as identified by DAPI staining, was evaluated as positive DAPI overlapping pixels. The number of overlapping pixels in NF-κB/p65 (green) images and DAPI (blue) images from each cellular nuclear area obtained from multiple images were determined and logged into an Excel spreadsheet. Observation for the subcellular localization of NF-κB/p65 in THP-1 cells was performed similarly as described previously with the exception that a special 96-well optics black plate with clear bottom polystyrene (Corning) was used. Cells were imaged on Nikon Eclipse Ti inverted microscope through an ×63 oil immersion objective lens (1.4 NA; PlanApo, Nikon) equipped with a 12-bit Coolsnap HQ cooled CCD camera (Roper Scientific). In addition to this, the expression of KOP receptor in differentiated THP-1 cells was also observed using a similar method as described above with the exception that mouse kappa opioid receptor monoclonal antibody (30 μg/mL) and secondary Alexa Fluor^®^ 647 goat anti-mouse IgG (H+L) polyclonal antibody (1:300) were used. The images of KOP receptor expression were observed using an inverted microscope at 20x magnification. The specificity of monoclonal KOP receptor used in the study was verified previously, showing no specific staining pattern in the absence of primary antibody.

### Human IL-1β and TNF-α bioassay

Differentiated THP-1 cells were seeded in 96-well microplates at a density of 4 x 10^5^cells/well and stimulated with LPS (1 μg/mL) (200 μL/well) to release IL-1β and TNF-α followed by incubation with DYN 1–17 and other fragments at different concentrations (0.1 μM, 1 nM and 10 pM) within a humidified atmosphere at 37°C and 5% CO_2_. Culture supernatants were collected after 24 hr of treatment period and added to a 384-well plate (1 μL/well) in duplicate. The level of human IL-1β and TNF-α secretion was quantified using AlphaLISA kit (Perkin Elmer, VIC, Australia) according to the manufacturer’s protocol. In brief, anti-IL-1β or anti-TNF-α conjugated to acceptor beads and biotinylated antibody were added into each well followed by one hour incubation at room temperature under subdued lighting. To detect the assay signal, streptavidin donor beads were added and the plate was incubated for 30 minutes at room temperature. The AlphaLISA signal was then read using an Enspire-Alpha 2390 Multilabel Plate Reader.

### Statistical analysis

All experiments were performed in triplicates and the data presented as the mean ± standard error of the mean (S.E.M.) from at least three independent experiments. Data calculations and comparisons between the groups were analysed with GraphPad Prism software (Version 6.07) using one-way ANOVA followed by post-hoc Bonferroni test. A significant difference between groups was defined as p ≤ 0.05.

## Results

### LPS stimulated the nuclear localization of NF-κB/p65 in differentiated THP-1 cells

**Fig 1:**
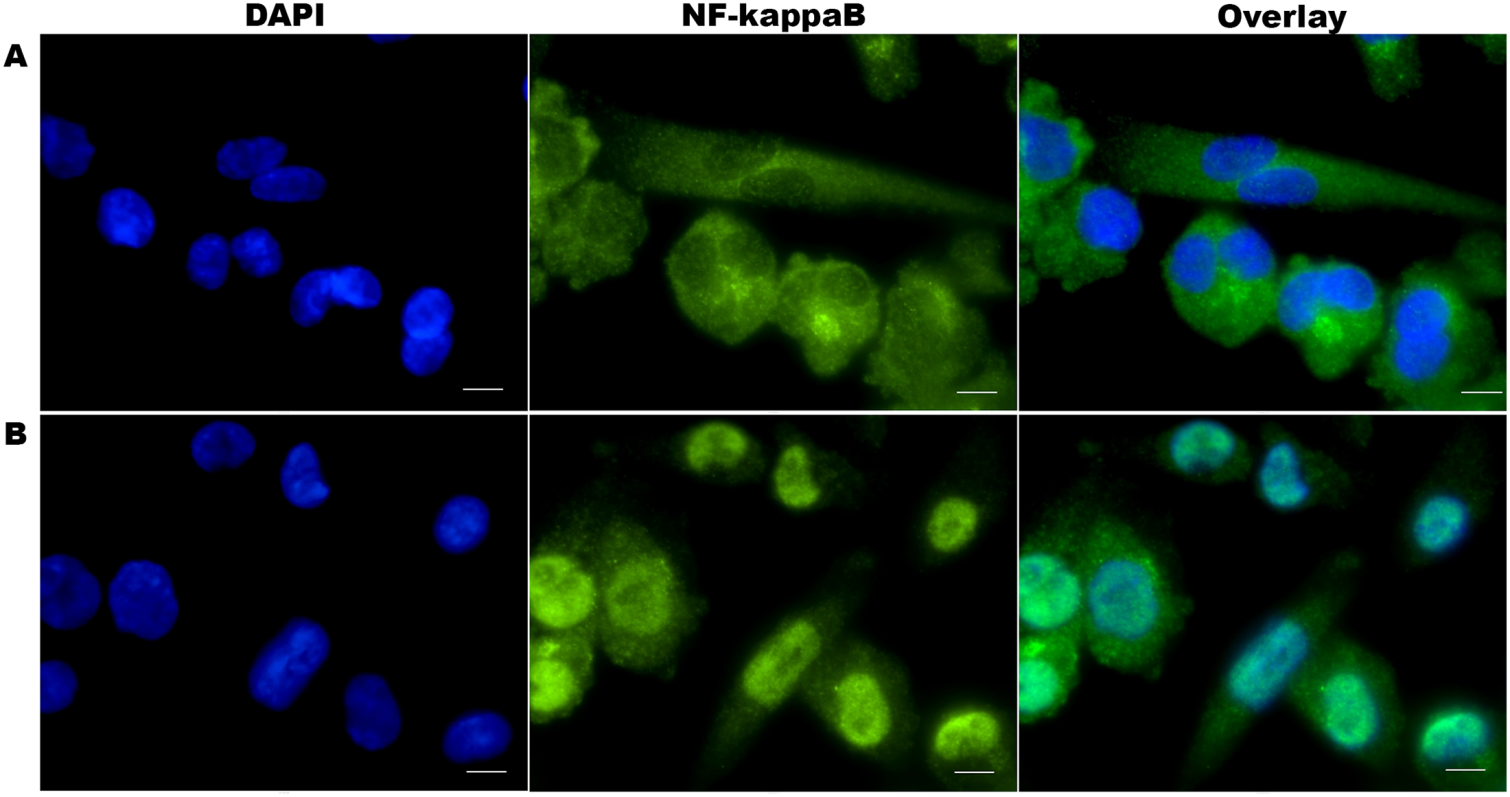
NF-κB/p65 nuclear localisation in differentiated THP-1 cells. Cells were fixed, permeabilised, stained with anti-NF-κB/p65 rabbit monoclonal antibody and visualised with Alexa Fluor^®^ 555 goat anti-rabbit IgG (green). The nuclei were counterstained with DAPI (blue). Microscopy images **A.** Inactive NF-κB/p65 proteins localisation in the cytoplasm of the non-stimulated THP-1 cells (top row) and **B.** Translocated NF-κB/p65 proteins into the nuclei of THP-1 cells following LPS stimulation (bottom row). Images were acquired for each fluorescence channel, using suitable TRITC and DAPI filters and a 63× objective. Scale bar: 10μm

The LPS-mediated NF-κB/p65 translocation in differentiated macrophages was observed qualitatively upon immunolabelling of differentiated THP-1 cells with NF-κB/p65 primary antibody and detected by Alexa Fluor^®^ 555 goat anti-rabbit IgG using TRITC filter and a 63× objective. The p65 subunit of NF-κB (green) was present predominantly in the cytoplasm of non-stimulated macrophages (Fig 1A). LPS stimulation resulted in strong nuclear labelling of NF-κB/p65 in stimulated cells (Fig 1B), reflecting the translocation of cytosolic NF-κB/p65 protein into the nuclei (blue). This result confirmed that LPS stimulation resulted in NF-κB/p65 translocation in PMA-differentiated THP-1 cells.

### DYN 1–17 and the N-terminal fragments inhibited NF-κB/p65 nuclear translocation in LPS-stimulated THP-1 cells

**Fig 2:**
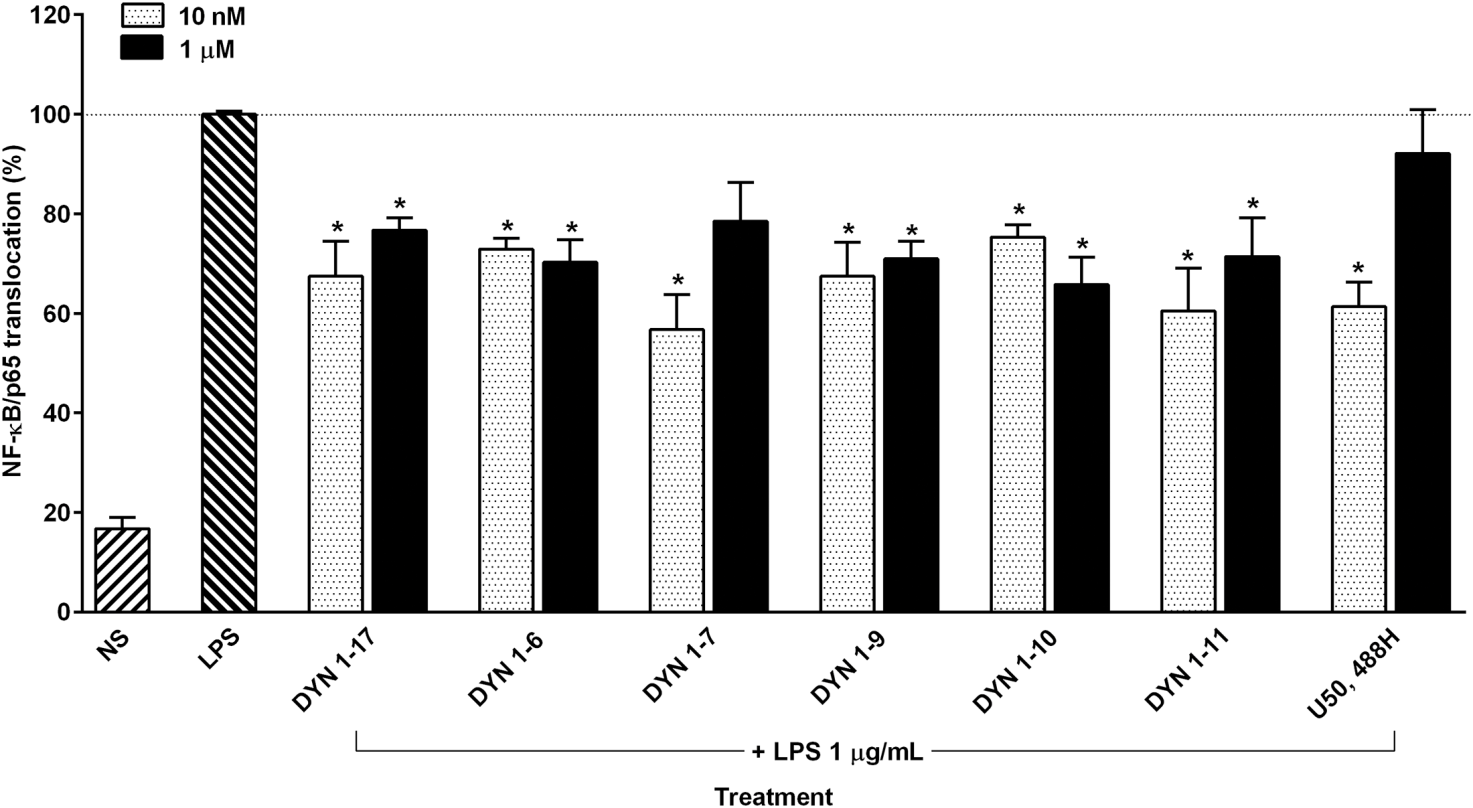
The attenuation of NF-κB/p65 nuclear translocation by DYN 1–17 and the N-terminal fragments in LPS-stimulated THP-1 cells. The LPS-stimulated THP-1 cells were treated with the N-terminal fragments of DYN 1–17 and U50,488H at 1 μM and 10 nM for an hour. The treated cells were fixed with paraformaldehyde (3.7%) and immunolabelled with primary anti-NF-κB/p65 monoclonal antibody and visualized using Alexa Fluor 555^®^ secondary antibody. DAPI staining was used to identify the nuclei. The nuclear translocation of NF-κB/p65 in each treatment group was assessed using the Image Xpress screening system. Non-stimulated THP-1 cells (NS) served as negative control. The NF-κB/p65 translocation percentage in each treatment group was normalised and expressed relative to LPS-stimulated control group. Data shown are the means ± S.E.M. of at least three independent experiments performed in triplicates, *p ≤ 0.05.

The ability of DYN 1–17 and its N-terminal fragments to inhibit the NF-κB/p65 nuclear translocation index in differentiated THP-1 cells was measured quantitatively using a high content screening approach. As shown in Fig 2, LPS induced greater than 50% of the NF-κB/p65 translocation in differentiated cells in comparison with the non-stimulated cells. Upon adding DYN 1–17 and the N-terminal fragments (DYN 1–6, 1–7, 1–9, 1–10, 1–11), NF-κB/p65 nuclear translocation was significantly inhibited between 20–40% at both high and low concentrations (1 μM and 10 nM, respectively) when compared to the LPS positive control group (p ≤0.05). This inhibitory effect was also observed in LPS-stimulated cells treated with U50,488H (1 μM and 10 nM), a selective KOP receptor agonist (p ≤0.05). Amongst these fragments, DYN 1–7 at 10 nM inhibited NF-κB/p65 translocation to the greatest extent, with around 44% inhibition relative to the LPS control group. This finding indicates that DYN 1–17 and the N-terminal DYN 1–17 fragments are involved in the attenuation of NF-κB/p65 nuclear translocation in LPS-stimulated THP-1 cells.

**Fig 3:**
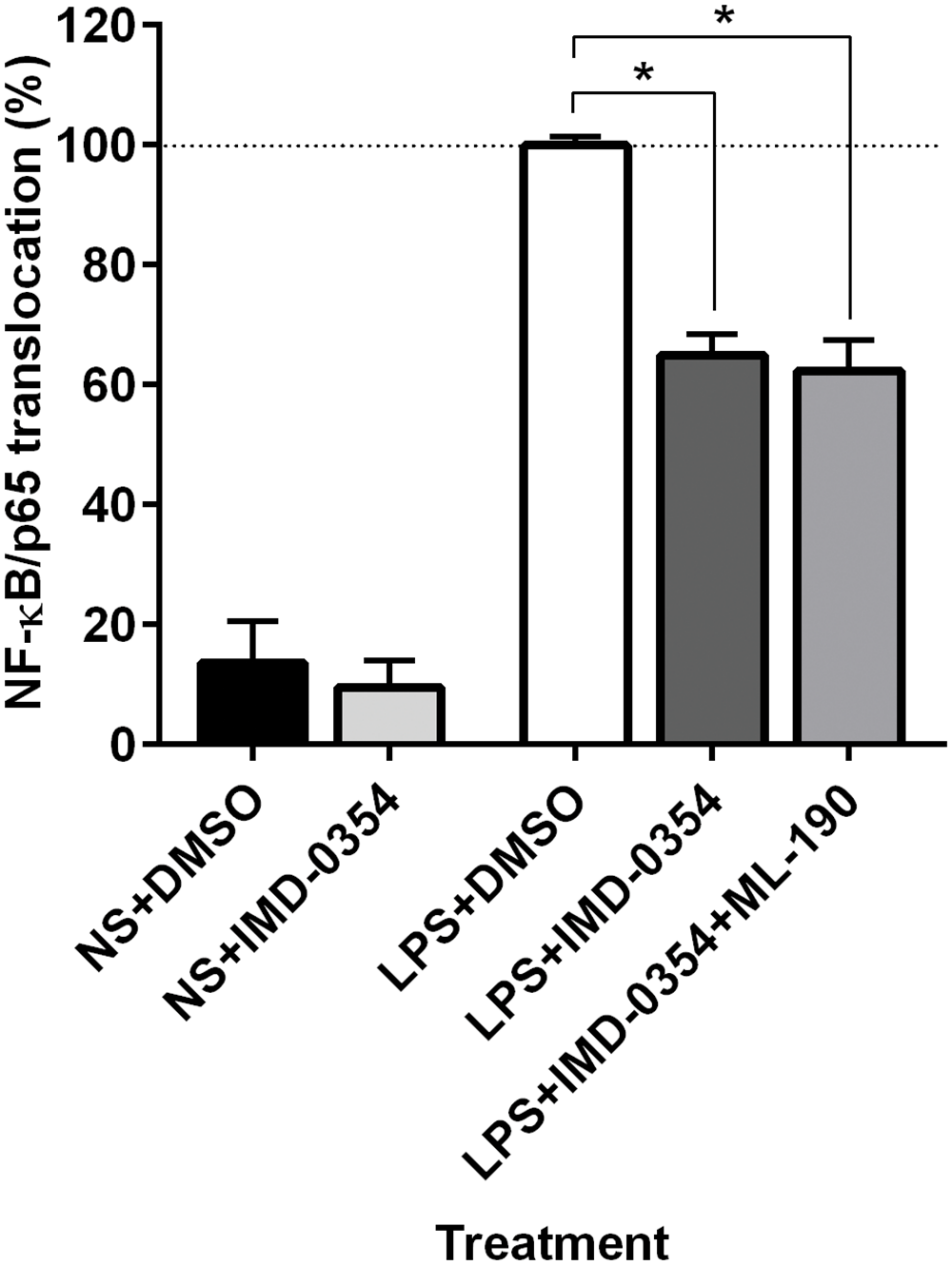
Effect of IMD-0354 on the NF-κB/p65 nuclear translocation in LPS-stimulated THP-1 cells. The LPS-stimulated THP-1 cells were treated with IMD-0354 (10 μM) and DMSO (0.1% v/v, as diluent control). The treated cells were fixed with paraformaldehyde (3.7%) and immunolabelled with primary anti-NF-κB/p65 rabbit monoclonal antibody and visualized using Alexa Fluor 555^®^ secondary antibody. DAPI staining was used to identify the nuclei. The nuclear translocation of NF-κB/p65 in each treatment group was assessed using the Image Xpress system. Non-stimulated THP-1 cells (NS), receiving similar treatment served as negative control. The percentage of NF-κB/p65 nuclear translocation in each treatment group was normalised and expressed relative to LPS-stimulated control group. Data represents means ± S.E.M. of at least three independent experiments performed in triplicates, *p ≤ 0.05.

DYN 1–17 and the N-terminal fragments, however, had no effect on NF-κB/p65 nuclear translocation in non-stimulated cells (S1 Fig). In addition, the MTT assay revealed that none of the N-terminal fragments of DYN 1–17 had any significant effect on the viability of THP-1 cells at both 10 nM and 1 μM in comparison with the relevant control group (S2 Fig). This finding therefore excludes non-specific cytotoxicity as a possible explanation for NF-κB/p65 inhibition. The differentiated THP-1 cells treated with LPS were also treated with IMD-0354, a selective IKK-2 inhibitor in order to check the functionality of our NF-κB/p65 nuclear translocation assay. IMD-0354 acts by blocking IKBα phosphorylation and thus prevents the induction of NF-κB/p65 nuclear translocation [23]. As shown in Fig 3, IMD-0354 produced a significant reduction in NF-κB/p65 translocation in comparison with the control group (DMSO 0.1% v/v as diluent control) (p ≤0.05). No significant difference for NF-κB/p65 nuclear translocation was observed in non-stimulated THP-1 cells treated with IMD-0354.

### The expression of KOP receptor in LPS-stimulated THP-1 cells

**Fig 4:**
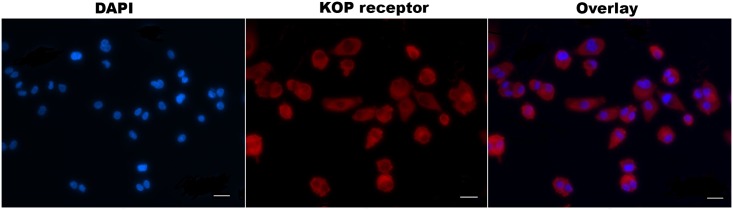
The expression of KOP receptor on LPS-stimulated THP-1 cells. Cells were fixed, permeabilised, immunolabelled with KOP receptor monoclonal antibody and visualised with Alexa Fluor 647^®^ goat anti-mouse IgG (red). The nuclei were counterstained with DAPI (blue). Images were acquired for each fluorescence channel, using suitable FITC and DAPI filters with 20 × objective. Scale bar: 25μm

DYN 1–17 has been reported to act primarily at the KOP receptor. As shown in Fig 4, the presence of KOP receptor expression was visualised on the cell surface of the LPS-stimulated differentiated THP-1 cells upon immunolabelling with a monoclonal antibody to KOP receptor. This observation further confirmed that membrane-bound KOP receptor is endogenously expressed in differentiated THP-1 cells.

### The involvement of KOP receptor in mediating the inhibitory effects of DYN 1–17 and the N-terminal fragments on NF-κB/p65 nuclear translocation in LPS-stimulated THP-1 cells

**Fig 5:**
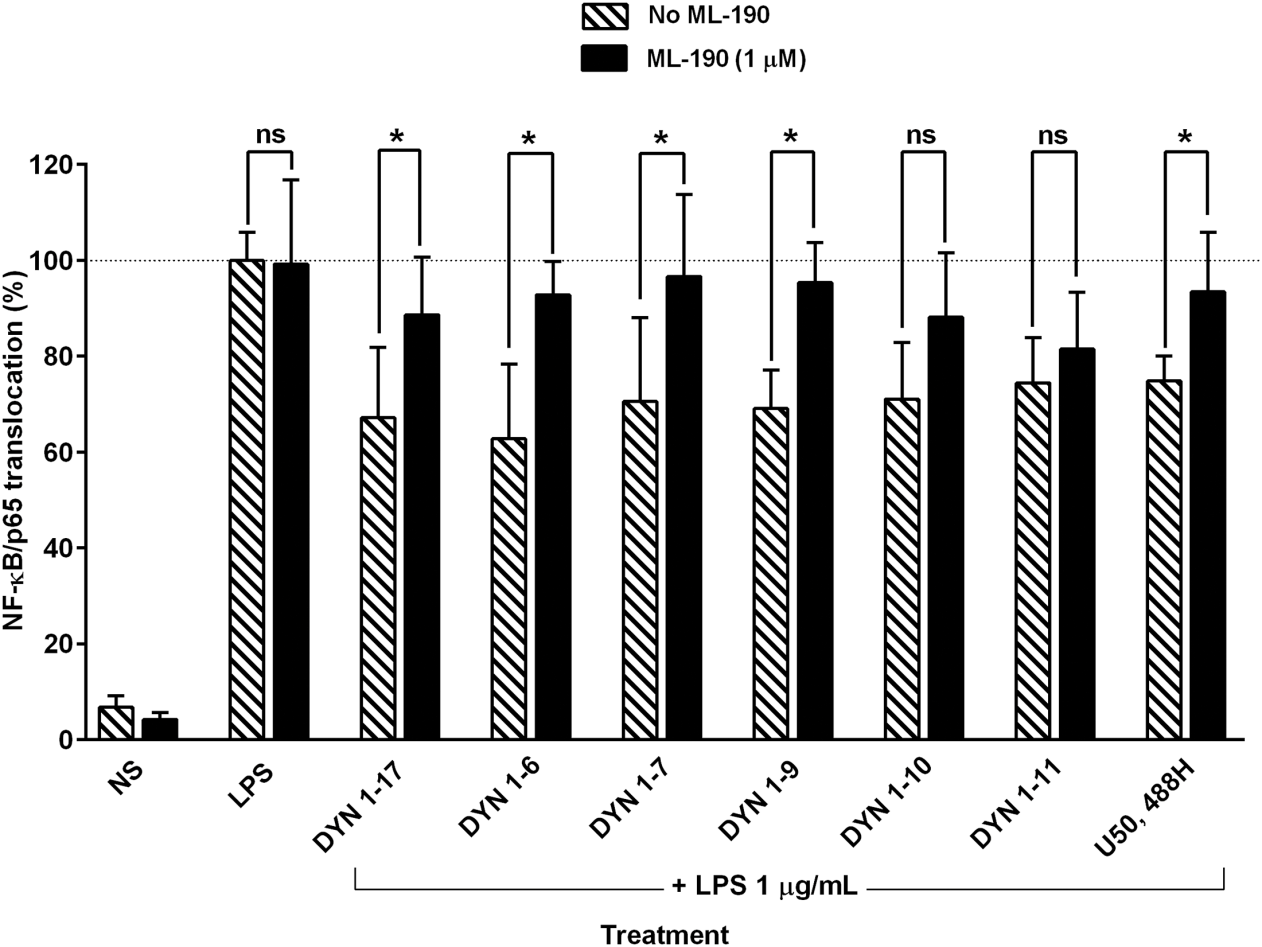
Effect of ML-190 on the attenuation of NF-κB/p65 nuclear translocation by DYN 1–17 and the N-terminal fragments in LPS-stimulated THP-1 cells. The LPS-stimulated THP-1 cells were pre-treated with ML-190, a KOP receptor antagonist (1 μM, 1 hour) followed by co-treatment of the DYN 1–17 and the N-terminal fragments (10 nM) or U50,488H (10 nM) with ML-190 (1 μM, 1 hour). The treated cells were fixed with paraformaldehyde (3.7%) and immunolabelled with primary anti-NF-κB/p65 rabbit monoclonal antibody and visualized using Alexa Fluor 555^®^ secondary antibody. DAPI staining was used to identify the nuclei. The nuclear translocation of NF-κB/p65 in each treatment group was assessed using the Image Xpress system. Non-stimulated THP-1 cells (NS) served as negative control. The percentage of NF-κB/p65 nuclear translocation in each treatment group was normalised and expressed relative to LPS-stimulated control group (contains DMSO 0.1% as diluent control). Data shown are the means ± S.E.M. of at least three independent experiments performed in triplicates, *p ≤ 0.05.

DYN 1–17 and U50,488H are KOP receptor agonists. To further investigate the involvement of KOP receptor in the attenuation of activated NF-κB/p65 translocation into the nucleus by the major biotransformation N-terminal fragments of DYN 1–17 and U50,488H (as a positive control), differentiated THP-1 cells were pre-treated with ML-190 (1 μM) followed by co-treatment of DYN 1–17 and other fragments (10 nM) or U50,488H (10 nM) with ML-190 (1 μM) in LPS-stimulated cells. DMSO 0.1% v/v served as the diluent control for ML-190. As shown in Fig 5, the inhibitory effects of DYN 1–17, DYN 1–6, DYN 1–7, DYN 1–9 and U50,488H on NF-κB/p65 nuclear translocation in LPS-stimulated cells were significantly reversed upon addition of ML-190 (p ≤0.05). However, no reversal effect was observed when ML-190 was co-treated with other N-terminal fragments (DYN 1–10 and DYN 1–11). Both LPS-activated cells treated with either DMSO or ML-190 alone exhibited similar activity on nuclear translocation as compared to the LPS-stimulated group (Fig 5). The results suggest that the inhibition of NF-κB/p65 nuclear translocation by DYN 1–17, DYN 1–6, DYN 1–7 and DYN 1–9 is mediated by the KOP receptor. The role of the KOP receptor was confirmed following ML-190 blockade of U50,488H mediated inhibition of NF-κB/p65 nuclear translocation (Fig 5).

### The effects of DYN 1–17 and the N-terminal fragments on the LPS-stimulated release of human IL-1β and TNF-α in differentiated THP-1 cells

**Fig 6:**
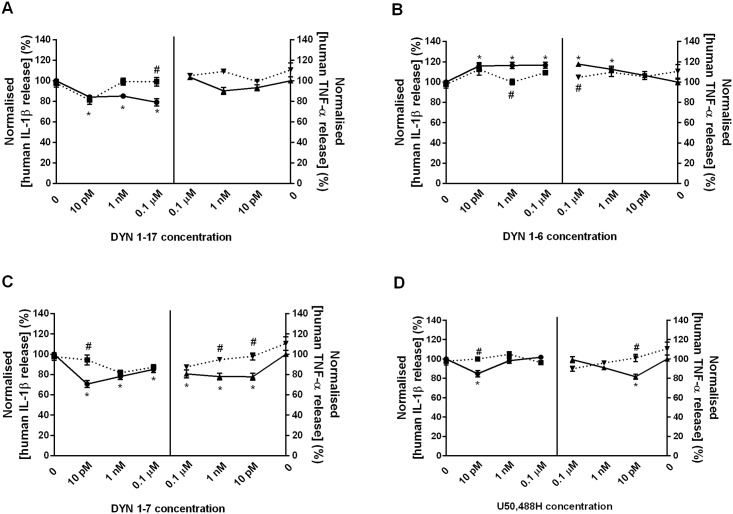
The modulation of DYN 1–17 and the N-terminal fragments on the release of IL-1β and TNF-α in LPS-stimulated THP-1 cells. The LPS-stimulated THP-1 cells were treated with the N-terminal fragments of DYN 1–17 and U50,488H at 0.1 μM, 1 nM and 10 pM for 24 hr. The culture supernatants were collected and IL-1β and TNF-α release was measured using IL-1β and TNF-α AlphaLISA kit, respectively. The AlphaLISA signal was read using an Enspire-Alpha 2390 Multilabel Plate Reader. Non-stimulated THP-1 cells (NS) served as negative control. The release of IL-1β and TNF-α in each treatment group was normalised and expressed relative to LPS-stimulated control group. Data shown are the means ± S.E.M. of at least three independent experiments performed in triplicates. Statistical significance as denoted by * and # represent the IL-1β and TNF-α release between peptide-treated (solid-line) and LPS-stimulated control group or ML-190-treated group (dotted-line), respectively, p ≤ 0.05.

In this study, the effect of DYN 1–17 and the N-terminal fragments (DYN 1–6, 1–7, 1–9, 1–10 and 1–11) on LPS-stimulated release of IL-1β and TNF-α was investigated at concentrations of 0.1 μM, 1 nM and 10 pM. LPS induced a significant increase of IL-1β and TNF-α levels at 24 hr, indicating that LPS activated THP-1 cells to release IL-1β and TNF-α in comparison with the non-stimulated cells (Fig 6). Treatment with DYN 1–17 resulted in a reduction of IL-1β release in a concentration-independent manner as compared to the LPS control group but no significant modulation on TNF-α release was observed (Fig 6A). Interestingly, DYN 1–7 significantly decreased the levels of both cytokines, with a 15–30% reduction in a concentration-dependent manner (p ≤0.05) when compared with LPS control group (Fig 6C). In contrast, administration of DYN 1–6 significantly augmented the release of IL-1β (at 0.1 μM, 1 nM and 10 pM) and TNF-α (at 0.1 μM and 1 nM) around 10–20% in comparison to LPS control group (Fig 6B). It is of note, however, that incubation with DYN 1–9, DYN 1–10 and DYN 1–11 did not have any significant modulation on the release of both IL-1β and TNF-α levels in LPS-stimulated THP-1 cells (S4 Fig). At 10 pM, the selective KOP receptor agonist, U50,488H inhibited the release of both IL-1β and TNF-α (Fig 6D) around 20% of the LPS control group (p ≤0.05).

To determine whether the changes to IL-1β and TNF-α release seen with the DYN fragments were mediated by the KOP receptor, ML-190 was used to block KOP receptor activity. Incubation with ML-190 (10 μM) alone did not significantly affect the LPS-stimulated IL-1β and TNF-α release in differentiated THP-1 cells (S3 Fig). Treatment with ML-190 significantly blocked the effects of DYN 1–17 (Fig 6A) and DYN 1–6 (Fig 6B) by 15–20% on the LPS-induced IL-1β release at 0.1 μM and 1 nM, respectively (p ≤0.05). Interestingly, ML-190 also partially reversed the inhibition of IL-1β release by DYN 1–7 (Fig 6C) and U50,488H (Fig 6D) at 10 pM (p ≤0.05). However, no reversal effect by ML-190 was found following modulation of DYN 1–17, DYN 1–6 and DYN 1–7 at other concentrations on LPS-stimulated IL-1β release. Further observations demonstrated that ML-190 blocked the augmentation of TNF-α release by DYN 1–6 (0.1 μM) (Fig 6B). Consistent with the IL-1β data, the inhibition of TNF-α release by DYN 1–7 (Fig 6C) and U50,488H (Fig 6D) at 10 pM was also significantly blocked by ML-190. Additionally, ML-190 reversed the inhibition of TNF-α by DYN 1–7 at 1 nM (Fig 6C).

Overall findings on the effects on DYN 1–17 and its N-terminal biotransformation fragments on NF-κB/p65 nuclear translocation and pro-inflammatory cytokine release were summarised in Table 1.

**Table 1:** The Effects of DYN 1–17 and Its N-terminal Biotransformation Fragments on LPS-stimulated NF-κB/p65 nuclear translocation.

Treatment	Inhibition of NF-κB/p65 nuclear translocation		Modulation of IL-1β release			Modulation of TNF-α release		
	10 nM	1 μM	10 pM	1 nM	0.1 μM	10 pM	1 nM	0.1 μM
DYN 1–17	√*	√	↓	↓	↓*	↔	↔	↔
DYN 1–6	√*	√	↑	↑*	↑	↔	↑	↑*
DYN 1–7	√*	X	↓*	↓	↓	↓*	↓*	↓
DYN 1–9	√*	√	↔	↔	↔	↔	↔	↔
DYN 1–10	√	√	↔	↔	↔	↔	↔	↔
DYN 1–11	√	√	↔	↔	↔	↔	↔	↔
U50,488H	√*	X	↓*	↔	↔	↓*	↔	↔

*significant antagonism with ML-190, p ≤ 0.05

↓ = inhibit, ↑ = enhance, ↔ = no changes, √ = yes, X = no

## Discussion

The NF-κB transcription factor is essential in regulating various inflammatory-related genes. Upon stimulation by LPS on the differentiated THP-1 cells, downstream activation of the NF-κB signalling pathway is triggered, leading to the production of various inflammatory cytokines such as IL-1β and TNF-α at the gene level [24]. Among the NF-κB transcription factor family of proteins, the most abundant complex found in mammalian species is the heterodimeric p65-p50. The p50 subunit has been suggested as a helper in DNA binding whereas the p65 subunit possesses a more important role as the activator of the transcriptional activity of NF-κB complex [25]. The NF-κB/p65 nuclear translocation assay performed in this study allows quantitative measurement on the degree of the translocation, hence enabling investigation of pharmacological modulation. This assay measures NF-κB/p65 redistribution from the cytoplasm to the nucleus and indirectly quantifies IκB degradation [26].

In the present study, the greatest inhibitory effect on NF-κB/p65 translocation was seen with DYN 1–7 at 10 nM, reducing translocation two-fold in comparison with the LPS control group. Other N-terminal fragments of DYN 1–17 elicited an inhibitory effect on NF-κB/p65 nuclear translocation at 1 μM and 10 nM, suggesting that these biotransformation fragments possess a potential immunomodulatory function during inflammation. Supporting this notion, N-terminal fragments of DYN 1–17 have been shown previously to modulate phagocytosis and tumouricidal activity in mouse [8, 27]. Notwithstanding that the affinity of opioid peptides for the opioid receptor is in the nanomolar range [16], the differential effects seen with concentration dependency can potentially be explained through the outcome of effects on multiple different mechanisms, with combined effects of the same signals.

Previous reports have also described that cytokine release is a consequence of extensive regulation of NF-κB transcription and translation processes [13, 28]. In this study, DYN 1–17 and select N-terminal biotransformation fragments (DYN 1–7 and DYN 1–6) modulated the release of IL-1β and TNF-α with the greatest reduction of both cytokines levels being observed following treatment with DYN 1–7 at low concentrations. The maximal inhibition of NF-κB/p65 translocation by DYN 1–7, which was around 40%, correlated with a 15–30% inhibition of IL-1β and TNF-α release. These findings are consistent with the modulation of NO and TNF-α release observed for DYN 1–17 and DYN 1–8 in mixed glia cells at subnanomolar concentrations [29]. It has also been reported that U50,488H at 0.1 nM suppressed the release of IL-1 and TNF-α in LPS-induced P388D1 macrophages, while at higher concentrations the effect was reduced [30]. Surprisingly, DYN 1–6 increased the release of both IL-1β and TNF-α whereas DYN 1–17 only modulated IL-1β and not TNF-α release, suggesting differential effects of these biotransformation fragments on cytokine modulation.

DYN 1–17 has been shown to bind to both opioid and non-opioid receptors [31, 32]. Many studies attribute the effects of DYN 1–17 largely to activity at the KOP receptor [33, 34]. Evidence indicates that the KOP receptor is expressed in cell lines of both neural and non-neural origin [35]. KOP receptor mRNA expression has been reported in THP-1 cells [36] and here we have verified the expression of the KOP receptor on the cell surface of LPS-induced differentiated THP-1 cells in this study. Up-regulation of KOP receptor expression concurrently with the release of DYN 1–17 in inflamed tissue has also been reported, indicating that inflammation directly influences opioid-mediated peripheral analgesia [37, 38].

The KOP receptor has also been demonstrated to mediate immune system function [39]. To determine whether the effect of selected fragments of DYN 1–17 on NF-κB/p65 translocation was mediated by KOP receptor, the selective KOP receptor antagonist, ML-190 was used. ML-190 is a newly discovered antagonist for KOP receptor that elicits much higher KOP receptor potency (IC_50_ = 120 nM in β-arrestin assay) over MOP and DOP receptors. In comparison with other selective KOP receptor antagonists such as GNTI, norBNI and JDTic, ML-190 possesses a promising potency and an improved selectivity for the KOP receptor (> 267-fold selective over that of MOP and DOP receptors) with a slightly cleaner binding profiles, making this drug suitable for studying KOP receptor distribution and internalisation [40]. Furthermore, ML-190 has a simplified structures that contains no stereochemical centres, highly modular and achiral compared to the other currently available KOP receptor antagonist with better solubility profile across the pH range tested. This antagonist is also readily synthesised following screening over a group of hit compounds due to its short and versatile synthetic route which allows the production of this compound on larger scale [41].

ML-190 significantly reversed the inhibitory effect of DYN 1–17 and N-terminal fragments (DYN 1–7, DYN 1–6 and DYN 1–9) on NF-κB/p65 translocation along with the KOP receptor selective agonist, U50,488H. This demonstrated that DYN 1–17 and N-terminal fragments (DYN 1–7, DYN 1–6 and DYN 1–9) suppress the translocation of cytosolic NF-κB/p65 protein into the nuclei of THP-1 cells through activation of KOP receptors. Consistent with our findings, activation of the KOP receptor in immune cells by DYN 1–17 and other derivatives negatively regulate the antigen-induced proliferation of T cells [42], antibody production by B cells [43], phagocytic ability [44] and also cytokine production by macrophages [45]. Furthermore, anti-tumour activity in peritoneal macrophages has been reported for DYN 1–17 and several N-terminal sub-fragments at KOP receptors [8, 46]. On the contrary, it was previously reported that DYN 1–17 and related fragments promote peritoneal macrophage phagocytosis, however this enhancement was not reversed by naloxone, suggesting that this immunomodulation is mediated via non-opioid receptors [27]. Furthermore, the difference of the methods and receptors used by Szabo et al. [44] and Ichinose et al. [27] for phagocytosis assay may explain the distinct responses of DYN 1–17 towards the phagocytic activity in mouse peritoneal macrophages.

The inhibition of NF-κB/p65 translocation by other longer N-terminal fragments (DYN 1–10 and DYN 1–11), however, was not reversed by ML-190; suggesting that, in this instance, the inhibitory effect of these fragments on NF-κB/p65 translocation could be mediated by other opioid receptors or by non-opioid receptors. The presence of a tyrosine residue on the first position of the amino acid chain allows binding between opioid peptides (except for orphanin) and their specific opioid receptors. Removal of the amino-terminal functional group abolishes the opioidergic activity of DYN 1–17 [47], with DYN 2–17 inactive at opioid receptors [46, 48].

The inhibition of both IL-1β and TNF-α release by DYN 1–7 occurred in a reverse concentration-dependent manner through interaction with KOP receptor which correlates with our prior NF-κB/p65 translocation findings. It is interesting to note that U50,488H, a selective KOP agonist, at 10 pM exhibited similar inhibitory effects to DYN 1–7 on the release of IL-1β and TNF-α in differentiated THP-1 cells and correlates with the previous findings for NF-κB/p65 translocation. Supporting this, a prior study has also reported the inhibitory effects of U50,488H at concentrations as low as 0.1 nM on the release of IL-1β and TNF-α in macrophage cell line P388D1 upon binding on KOP receptor. This effect was similarly shown to be mediated via the KOP receptor by reversal with norbinaltorphimine (nor-BNI), a kappa-selective antagonist [30].

The discrepancy between effects of DYN 1–17 and DYN 1–6 in comparison with DYN 1–7 on IL-1ß and TNF-α release suggest differential effects of KOP receptors on cytokine modulation. Similarly, previous studies have reported different responses produced by ligands that are active at the same receptor site [49]. The differential effects exhibited by KOP receptor agonists in mediating KOP receptor-mediated antinociception has been reported previously. At low concentrations, a selective KOP receptor subtype 3 agonist, nalorphine, significantly blocked morphine analgesia, however at a higher concentration, the agonist produced complete analgesia [50]. The KOP selective agonist U69,593 was found to elicit a higher analgesic response in comparison to U50,488H (95% vs 60%) [51]. Furthermore, the differences in signalling mechanisms of the KOP receptor agonists could be due to the opioid receptor heterodimerisation as shown by the synergistic binding of highly selective agonists by kappa-delta receptor heterodimers that further potentiates the signal transduction which are distinct from those of either receptor [52].

In addition, despite of the common structural elements shared between the non-peptide kappa selective ligands and the naturally occurring kappa opioid peptides, both classes showed differential modulation on immune functions. This discrepancy is not clear; however several possibilities may be suggested. Firstly, the complex structure with multiple stereocenters of the non-peptide KOP receptor agonists in comparison with the kappa opioid peptides results in differential regulation of the KOP receptor due to the different receptor conformational changes induced by both classes of ligands [40, 53]. Secondly, both classes of ligand may have a distinct regulation of calcium signalling in immune cells. Calcium signalling has been denoted as one of the second messenger systems that play an important role in opioid-mediated immune activation and regulation [54]. Supporting this, an earlier study has reported that both dynorphin and U50, 488H regulate the intracellular calcium homeostasis differently in guinea pig cerebellar synaptomes [55]. Finally, the kappa opioid peptides may have a tendency to bind to other opioid receptors albeit with low affinity whereas the non-peptide kappa ligands such as U50, 488H selectively bind to KOP receptor only [30], which defines the diverse immune regulatory pathways. Taken together, all these possibilities may explain the diverse immune regulatory pathways by both classes of KOP receptor ligands due to their distinct sensitivities on KOP receptor in producing their biological effects in immune cells.

In conclusion, DYN 1–17 and select N-terminal fragments modulate the nuclear translocation of NF-κB/p65 protein and subsequent modulation of downstream pro-inflammatory cytokine release. Based on structural-specificity relationship of the fragments, we suggest that N-terminal fragments, comprising seven amino acids in the peptide sequence, are acting through the KOP receptor—as demonstrated by DYN 1–7, that effectively inhibits NF-κB/p65 translocation and downstream release of inflammatory IL-1β and TNF-α cytokines through KOP receptor. It is plausible that the involvement of non-KOP receptor mechanisms mediate the effects at different concentrations as well as for other N-terminal fragments in the regulation of NF-κB/p65 signal and cytokine release in differentiated THP-1 cells, which warrants further investigation.

## Supporting Information

S1 Data Numerical data used in preparation of Figs 2, 3, 5, 6A–6D and S1, S2, S3A and S3B, S4A, S4B and S4C Figs. (XLSX)

S1 Fig The absence of NF-κB/p65 nuclear translocation by DYN 1–17 and the N-terminal fragments in non-stimulated THP-1 cells. The non-stimulated THP-1 cells were treated with the N-terminal fragments of DYN 1–17 and U50,488H at 1 μM and 10 nM for an hour. The treated cells were fixed with paraformaldehyde (3.7%) and immunolabelled with primary anti-NF-κB/p65 monoclonal antibody and visualized using Alexa Fluor 555^®^ secondary antibody. DAPI staining was used to identify the nuclei. The nuclear translocation of NF-κB/p65 in each treatment group was assessed using the Image Xpress screening system. Non-stimulated THP-1 cells (NS) served as negative control. The NF-κB/p65 translocation percentage in each treatment group was normalised and expressed relative to LPS-stimulated control group. Data shown are the means ± S.E.M. of at least three independent experiments performed in triplicates.
(TIF)

S2 Fig Cell viability of LPS-induced THP-1 cells following treatment with DYN 1–17 and the N-terminal fragments. Cell viability of LPS-induced THP-1 cells was determined by the MTT assay. Cells were exposed to the peptides for 24 hr and cells were treated with MTT and incubated for a further 4 hr. The metabolite formazan was measured using a microplate reader. Data shown are the means ± S.E.M. of at least three independent experiments performed in triplicates.
(TIF)

S3 Fig Effect of ML-190 and DMSO on the release of A. IL-1β and B. TNF-α in LPS-stimulated THP-1 cells. The LPS-stimulated THP-1 cells were treated with ML-190 (10 μM) and DMSO (0.1%) for 24 hr. The culture supernatants were collected and IL-1β and TNF-α release was measured using IL-1β and TNF-α AlphaLISA kit, respectively. The AlphaLISA signal was read using an Enspire-Alpha 2390 Multilabel Plate Reader. Non-stimulated THP-1 cells (NS) served as negative control. The release of IL-1β and TNF-α in each treatment group was normalised and expressed relative to LPS-stimulated control group. Data shown are the means ± S.E.M. of at least three independent experiments performed in triplicates.
(TIF)

S4 Fig Effect of DYN 1–9, DYN 1–10 and DYN 1–11 on the release of IL-1β and TNF-α in LPS-stimulated THP-1 cells. The LPS-stimulated THP-1 cells were treated with DYN 1–9, DYN 1–10 and DYN 1–11 at 0.1 μM, 1 nM and 10 pM for 24 hr. The culture supernatants were collected and IL-1β and TNF-α release was measured using IL-1β and TNF-α AlphaLISA kit, respectively. The AlphaLISA signal was read using an Enspire-Alpha 2390 Multilabel Plate Reader. Non-stimulated THP-1 cells (NS) served as negative control. The release of IL-1β and TNF-α in each treatment group was normalised and expressed relative to LPS-stimulated control group. Data shown are the means ± S.E.M. of at least three independent experiments performed in triplicates.
(TIF)
